# Putative azithromycin resistance mutations in *Chlamydia trachomatis* are globally distributed but arose before azithromycin was discovered

**DOI:** 10.1128/aac.01708-25

**Published:** 2026-01-26

**Authors:** Parul Sharma, Deborah Dean, Timothy D. Read

**Affiliations:** 1 Division of Infectious Diseases, Department of Medicine, Emory University School of Medicine 1371, Atlanta, Georgia, USA; 2 Division of Infectious Diseases and Global Health, Departments of Medicine and Pediatrics, University of California San Francisco School of Medicine 12224 , San Francisco, California, USA; 3 Department of Bioengineering, Joint Program, University of California San Francisco and University of California Berkeley 8785, San Francisco, California, USA; Columbia University Irving Medical Center, New York, New York, USA

**Keywords:** genomic surveillance, azithromycin, antimicrobial resistance, *Chlamydia trachomatis*

## Abstract

Azithromycin is widely used to treat *Chlamydia trachomatis* infections, yet the extent of resistance to the drug across the species has not been addressed. We surveyed mutations and substitutions linked to putative azithromycin resistance across 1,349 high-quality *C. trachomatis* genomes. Mutations in the *rpl*V gene encoding three non-synonymous substitutions, compared with the canonical *C. trachomatis* reference strain D//TW-3/Cx sequence, were found to be common but largely conserved within phylogenetic lineages causing prevalent urogenital and anorectal infections and lymphogranuloma venereum. However, no mutations were identified in the ocular lineage. Time-scaled phylogenetic analysis suggested that these mutations predate the clinical introduction of azithromycin. In contrast, no consistent resistance-associated patterns were observed in *23S* rRNA or *rpl*D genes. This large-scale genomic surveillance provides critical insights into the evolutionary trends of putative azithromycin resistance in *C. trachomatis* and underscores the importance of integrating genomic monitoring with phenotypic susceptibility testing to accurately assess and manage antimicrobial resistance.

## INTRODUCTION

*Chlamydia trachomatis* is an obligate intracellular pathogen and the most common cause of bacterial sexually transmitted infections (STIs) worldwide. Ocular strains of *C. trachomatis* cause trachoma, the leading cause of infectious blindness, particularly among women due to their increased exposure to children who are reservoirs of infection in trachoma-endemic regions (1). In STIs, treatment commonly involves antibiotics, such as doxycycline, administered twice a day over 7 days or azithromycin as a single dose (2). Recent clinical evidence indicates that doxycycline is more effective than azithromycin for treating rectal chlamydial infections (3). For trachoma, topical tetracycline ointment or oral azithromycin has historically been the preferred option (4). Azithromycin is the antibiotic of choice in many mass drug administration (MDA) campaigns implemented under the World Health Organization (WHO) Surgery, Antibiotics, Facial cleanliness, and Environmental improvement (SAFE) strategy, aimed at eliminating blinding trachoma (5). Despite large-scale efforts, elimination targets have not been fully achieved, and several countries, particularly in sub-Saharan Africa, such as Ethiopia and Sudan, remain endemic. In recognition of these challenges, the WHO extended its global elimination goal for blinding trachoma from 2020 to 2030 (6, 7).

Azithromycin is an azalide, a sub-class of macrolide antibiotics (8). It was discovered in 1980, patented in 1981, and its widespread use began after 1991 when it was launched by Pfizer (9). Since then, it has become one of the most widely prescribed antibiotics for bacterial infections, including *C. trachomatis*, due to its broad-spectrum activity, tissue penetration and long half-life, and patient compliance advantages (10, 11). However, its extensive use has also raised concerns about the potential for emerging resistance.

Several STI studies have reported *C. trachomatis* treatment failures with azithromycin (12–14). These failures are often attributed to either reinfection of another *C. trachomatis* strain after clearance of the initial infection or persistence of the initial infection. Persistent infections have often been attributed to the development of *C. trachomatis* antibiotic resistance (14, 15). Several studies have identified point mutations in 23S rRNA, *rplV,* and *rplD* genes as potentially linked to macrolide resistance in *C. trachomatis* (16–18). In *rpl*V, encoding the L22 protein, mutations resulting in G52S, R65C, and V77A substitutions were detected in isolates from patients with azithromycin treatment failure (19, 20), although the minimum inhibitory concentrations (MICs) were within the sensitive range. The same variants were observed in Russian urogenital isolates in both macrolide-resistant and sensitive strains (21), and among endocervical, vaginal, and rectal samples in Fiji (22). For RplD, the L4 protein, studies reported P109L, P151A (20), and Q66K substitutions (16), while other studies (19, 23) did not detect *rpl*D mutations in clinical isolates. 23S rRNA gene mutations A2057G, A2058C, A2059G, and T2611C have been associated with resistance, although their presence and role remain inconsistent across studies (20, 21, 24, 25). Notably, all the above 23S rRNA mutations are also listed in the NCBI Reference Gene Catalog (26), a public database that compiles known antimicrobial resistance genes and associated mutations across diverse pathogens. Similarly, the Comprehensive Antibiotic Resistance Database (CARD; 27) serves as another curated resource, detailing resistance determinants, mechanisms, and mutation-based associations. The latest version of CARD (accessed October 2025) specifically documents *23S* rRNA mutations linked to azithromycin resistance in *C. trachomatis*, while the NCBI catalog lists *rpl*D, *rpl*V, and *23S* rRNA mutations reported across multiple bacterial species.

In this study, we analyze the patterns of DNA mutations in the 23S rRNA gene and DNA mutations and amino acid substitutions in L22 and L4 associated with azithromycin resistance across a globally distributed collection of 1,349 *C*. *trachomatis* genomes from public databases. Our aim was to characterize the prevalence, lineage specificity, and evolutionary patterns of putative azithromycin resistance variants.

## MATERIALS AND METHODS

### Compiling and pre-processing genomic data

*C. trachomatis* genomes were compiled from multiple sources, including the NCBI Assembly database (accessed March 2025), AllTheBacteria database (version 0.2 with the incremental release from 08 to 2024) (28), published reference genomes (29), and available NCBI SRA samples. For NCBI SRA samples, raw reads were down-sampled to achieve even coverage using the bbnorm.sh script from BBMap (v39.01) (30), followed by assembly with SPAdes (v4.0.0) (31) using default parameters. Quality filtration was applied across the complete data set of genomes from all sources using checkM (v1.2.3) (32) retaining only genomes with more than 98% completeness, less than 6% contamination, and less than 2,000 contigs. Metadata for genomes in this study in Table S1.

### Identifying azithromycin resistance-associated mutations

A list of point mutations and substitutions linked to macrolide resistance was obtained from the NCBI Reference Gene Catalog (accessed March 2025) (26) and CARD (27). Additional candidate mutations reported in the literature for azithromycin resistance in *C. trachomatis* were also compiled (Table 1). To ensure comparability, all mutations were converted to *C. trachomatis* numbering by alignment to *C. trachomatis* reference strain D/UW-3/Cx (NCBI Accession ID: NC_000117.1) (33).

**TABLE 1:** List of azithromycin-associated 23S mutations in public databases

Source	Allele	Reference	Reference species	CT-numbering	Detected
NCBI	a2074c/a2074g/a2074t	NC_022347.1, NC_002163.1	*Campylobacter coli,* *Campylobacter jejuni*	a1879c/a1879g/a1879t	Yes (a->g)
	a2075g	NC_022347.1, NC_002163.1	*Campylobacter coli,* *Campylobacter jejuni*	a1880g	
	a2059g	NC_002946.2	*Neisseria gonorrhoeae*	a2036g	Yes
	a2058g / a2058t	NC_004431.1	*Escherichia coli*	a2038g	
	a2114g	NZ_CP018347.1	*Streptococcus pneumoniae*	a2449g	
	a2115g	NZ_CP018347.1	*Streptococcus pneumoniae*	a2450g	
	c2627a	NC_022347.1, NC_002163.1	*Campylobacter coli,* *Campylobacter jejuni*	c2592a	
	c2611t	NC_002946.2, NC_004431.1	*Neisseria gonorrhoeae,* *Escherichia coli*	c2592t / c2592g	Yes (c->t)
	g2032t	NC_004431.1	*Escherichia coli*	g2012t	
	c2630a/c2630g	NZ_CP018347.1	*Streptococcus pneumoniae*	na^*a*^	
	t2609c	NC_004431.1	*Escherichia coli*	t2590c	
	t754a	NC_004431.1	*Escherichia coli*	t740a	Yes (t->g)
CARD	c196a	NR_076160.1	*Chlamydia trachomatis*	c196a	
	a2107c/a2107g	NR_076160.1	*Chlamydia trachomatis*	a2107c / a2107g	
	a2109g	NR_076160.1	*Chlamydia trachomatis*	a2109g	
	a2663c / t2663c	NR_076160.1	*Chlamydia trachomatis*	a2663c / t2663c	

^*a*^ na, not aligned. Detected mutations were present in more than 99% of the isolates. *C. trachomatis*-numbering was determined by aligning the reference sequence against *C. trachomatis* reference NC_000117.1.

All genomes in the data set were re-annotated using Bakta (v1.9.2) (34) to minimize annotation heterogeneity, and sequences corresponding to the 23S rRNA, *rpl*V, and *rpl*D genes were extracted. Each gene set was aligned to the reference sequence using MAFFT (v7.526) (35), and variants were identified relative to the reference coordinates. Mutations were detected and summarized using custom python scripts (available on GitHub: https://github.com/parul-sharma/CT-AMR).

### Phylogenetic trees and time-scaled analysis

Core-genome genes were identified for all annotated genomes in the data set using PIRATE (v.1.0.5) (36) under a single workflow. The resulting core gene alignment, composed of 853 genes 0.83Mb in length, was used as input for IQ-TREE2 (version 2.3.0) (37) to construct a maximum-likelihood phylogenetic tree with automated model selection. A similar phylogenetic tree was obtained for the subset of genomes (138 out of 1,354) with associated dates of isolation. For these dated strains, dates of isolation were added using the “–date” parameter to supply temporal metadata to the IQ-TREE run. The resulting tree was subsequently used as input for BEAST (v2.7.7) (38) to perform time-scaled phylogenetic inference.

Using BEAST, we evaluated multiple models for phylogenetic inference. All models used a strict molecular clock but differed in population models: (i) Bayesian Skyline; (ii) Bayesian Skyline with HKY nucleotide substitution; (iii) exponential growth; (iv) constant population size; and (v) extended Bayesian Skyline with invariant sites. All models produced broadly similar topologies and divergence times (Supplementary Data). For clarity, the results presented here are based on the strict molecular clock with an exponential growth population model. Random Local Clock models were not attempted, as the low overall divergence of *C. trachomatis* sequences would provide insufficient signal to reliably estimate lineage-specific rate variation.

### Recombination and visualizations

Recombination analysis was performed on the core genome alignment (described above) using ClonalFrameML (v1.13) (39) with default parameters. The resulting importation status file was processed with a custom Python script (available on GitHub https://github.com/parul-sharma/CT-AMR) to identify recombination regions. Briefly, the genome was divided into 1,000 bp windows, the total number of recombination events within each window was counted, and windows with elevated recombination activity (more than 20 recombination events) were designated as recombination “hotspots.”

To investigate whether resistance-associated loci showed phylogenetic patterns distinct from the core genome, we constructed individual gene trees for the 23S, *rpl*V, and *rpl*D sequences. These were then compared with the core-genome phylogeny using tanglegrams, allowing us to check congruence in tree structure and evaluate potential evidence of recombination or lineage-specific inheritance at these loci. All visualizations were generated in R, with trees constructed using ggtree (v3.13.0) (40), time-scaled trees in combination with treeio (v1.28.0) (41), and tanglegram comparisons performed using phytools (v2.4-4) (42). Supporting R scripts are available in the GitHub repository.

## RESULTS

### Putative-resistance mutations in the *rplV* gene are lineage specific

We examined resistance-associated mutations in our data set of 1,349 high-quality *C. trachomatis* genomes (Table S1). A core-genome phylogeny divided the genomes into four major lineages, consistent with prior studies: lymphogranuloma venereum (LGV), ocular, prevalent urogenital/anorectal (P-UA), and non-prevalent urogenital/anorectal (NP-UA), reflecting their disease tropisms (43–45). All substitutions were called in relation to the canonical reference D/UW-3/Cx sequence (NC_000117.1), an NP-UA genome (33).

**Fig 1:**
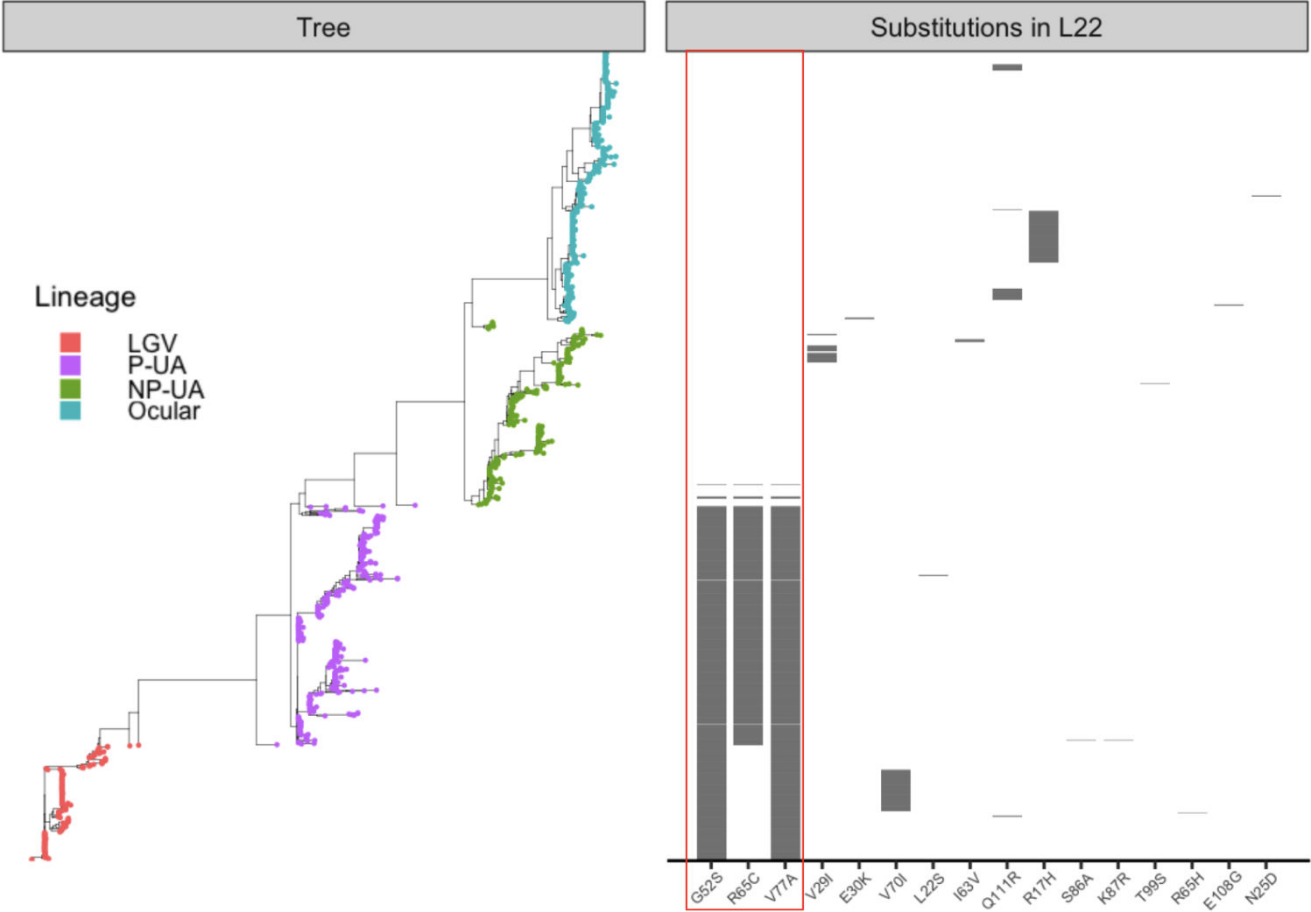
Distribution of L22 substitutions across genomes. Genomes are ordered according to core-gene phylogeny (left) with tips colored by *C. trachomatis* lineage (LGV, ocular, P-UA, NP-UA). The panel on the right shows a heatmap of the 16 L22 substitutions, marked along the x-axis, with gray tiles indicating presence and empty spaces indicating absence across samples. The three azithromycin-associated substitutions are marked with a red box.

We first focused on the three amino acid substitutions in L22: G52S, R65C, and V77A that have been associated with azithromycin resistance in *C. trachomatis* (19, 21). In each case, we found that the substitution was caused by a single mutation (g151a, c190t, and t227c, respectively). These substitutions showed strong lineage specificity (Fig. 1). All LGV genomes (191/191, 100%) carried G52S and V77A, while nearly all P-UA genomes (399/404, 98.8%) harbored the complete set of triple substitutions (G52S, R65C, and V77A). In contrast, these putative resistance-associated substitutions were completely absent from ocular genomes (0/450) and were rare in NP-UA genomes (4/304, 1.3%). In the rare NP-UA genomes with the G52S, R65C, and V77A substitutions, the sequence was identical to the P-UA allele, suggesting exchange by homologous recombination. A similar pattern in reverse was seen in the rare P-UA genomes missing these mutations (Table S2).

We next broadened our analysis to identify additional *rpl*V mutations that resulted in amino acid substitutions relative to the reference D/UW-3/Cx protein sequence (accession ID: NC_000117.1) across the data set. Thirteen other substitutions were detected, most at low frequencies and restricted to specific lineages, with the only exception being the Q111R substitution present in few of both Ocular (28/450, 6.2%) and LGV (2/191, 1.0%) genomes (Fig. 1). In the P-UA lineage, S86A and K87R co-occurred in a single genome, while L22S was detected in another. Among ocular genomes, R17H was the most common secondary variant (86/450, 19.1%), followed by Q111R (28/450, 6.2%), with several others present at very low frequencies (N25D, E30K, and E108G, each in ≤2 genomes). In NP-UA genomes, V29I was observed in 26/304 (8.5%), I63V in 4/304 (1.3%), and T99S in a single genome. Within LGV genomes, Q111R was present in 2/191 (1.0%), while V70I was more frequent (68/191, 35.8%); R65H occurred only once.

Together, these findings demonstrated that the putative azithromycin-associated *rplV* mutations were tightly clustered in P-UA and LGV lineages. The absence of these variants in ocular genomes likely reflects their long-standing evolutionary separation from P-UA and LGV lineages. There were other substitutions, but aside from Q111R, there was no evidence of homoplasy. There was also evidence of gene conversion events in the P-UA and NP-UA lineage strains.

### L22 resistance-associated substitutions predate the widespread clinical use of azithromycin

**Fig 2:**
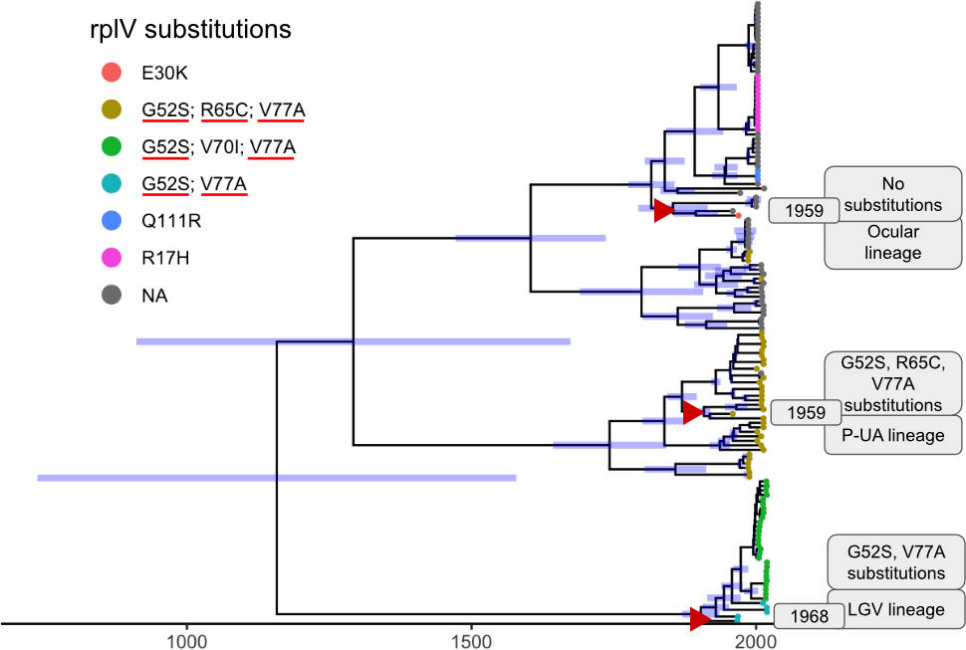
Time-scaled phylogeny constructed using BEAST. Branch lengths are proportional to time (in years), and the 95% highest posterior density (HPD) intervals of node heights are indicated as blue bars. Tip labels annotated by color representing *rpl*V substitutions as per the legend with azithromycin-associated substitutions marked with red underline. The three oldest genomes (highlighted with red triangles) are annotated with metadata, including year of isolation, lineage, and presence of *rpl*V substitutions.

For a subset of 138 (10.2%) of the 1,349 genomes, metadata on the year of isolation were available (Table S1). The most recent samples in our data set were from 2019. Genome SAMEA767935 (P-UA lineage, E/Bour *omp*A type), collected in 1959 (46), carried all three canonical L22 resistance-associated substitutions (G52S, R65C, and V77A). Another 1959 genome, SAMEA1973344 (Ocular lineage, C/TW-3/OT *omp*A type), had no substitutions in L22. The second-oldest genome, SAMEA767923 (LGV lineage, L3/404 *omp*A type), collected in 1967, carried the double mutations (G52S and V77A). The presence of resistance-associated *rplV* mutations in genomes dating from 1959 and 1967 indicated that these mutations arose prior to the widespread clinical use of this antibiotic after 1991. Time-scaled phylogenetic analysis (Fig. 2) further supported this observation. Using the strict molecular clock with an exponential growth population model, we estimated a 95% likelihood that genomes carrying the triple mutations diverged between 1,016 and 1,779, while those with the double mutations diverged between 850 and 1,695.

### Patterns of mutations in *rpl*D and 23S rRNA

**Fig 3:**
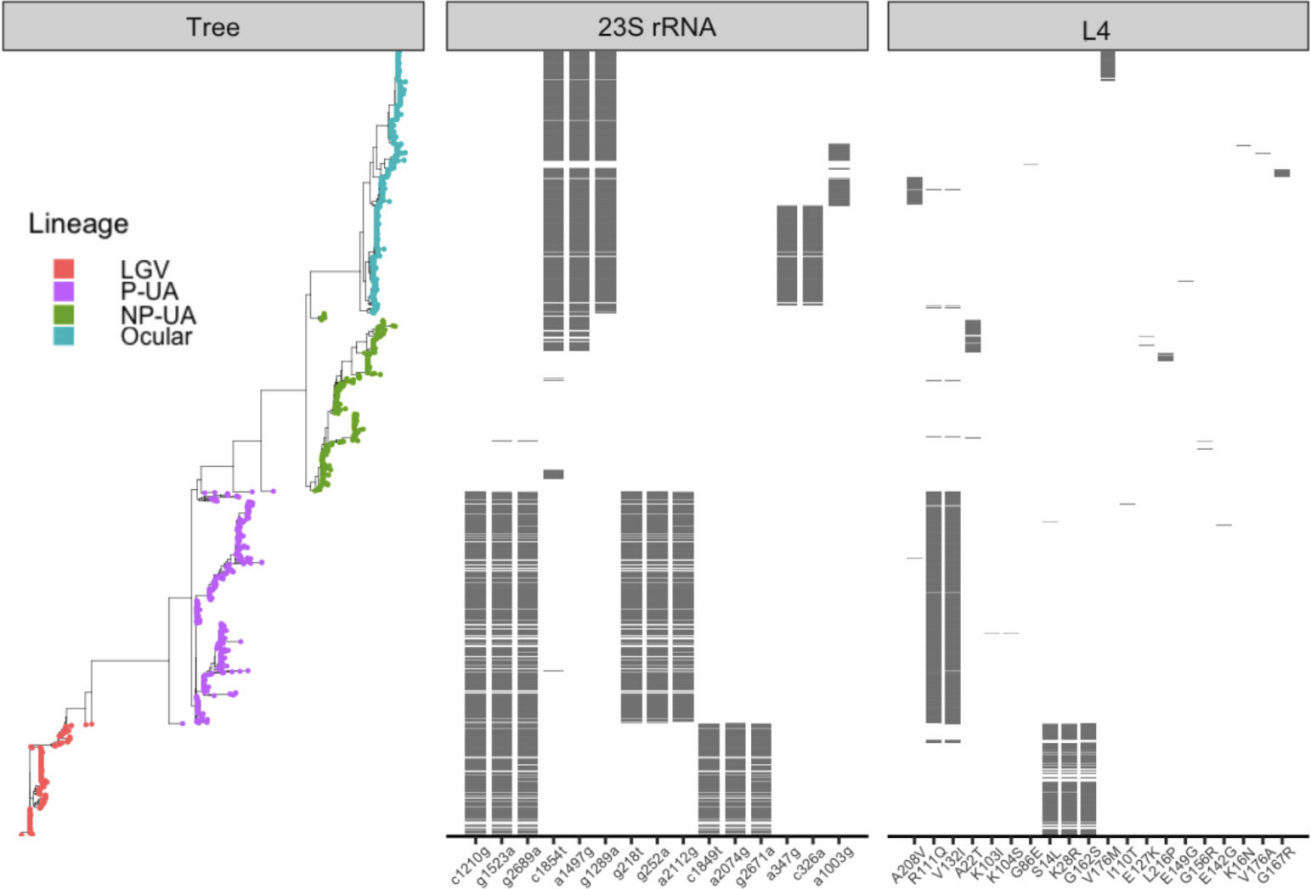
Distribution of mutations in 23S rRNA and substitutions in L4 across genomes. Genomes are ordered by core-gene phylogeny (left), with tree tips colored by genome lineage. The first panel shows the 15 most frequent 23S rRNA mutations; the second panel shows all L4 substitutions. In both panels, the x-axis lists individual mutations or substitutions.

The three L4 protein substitutions previously reported as azithromycin resistance-associated (P109L, P151A [20], and Q66K [16]) were not found across the 1,354 *C*. *trachomatis* genomes in this study. Due to the high sequence divergence of the *rpl*D gene across bacterial species, none of the putative resistance-associated substitutions listed in the NCBI Reference Gene Catalog could be identified in our data set, as protein sequences from other species did not align with the *C. trachomatis* L4 protein (Table S3). A small number of substitutions were observed within the protein, most of which were lineage-specific (Fig. 3). Notably, two homoplasic substitutions—R111Q and V132I—were predominant in P-UA genomes (399/404, 98.8%), though they also appeared in a few NP-UA (*n* = 2), Ocular (*n* = 3), and LGV (*n* = 6) genomes. These variants were absent in five P-UA genomes and partially missing in three others, missing R111Q in two and V132I in one (Table S4).

Similarly, examination of the 23S rRNA sequence showed no obvious correlation with previously reported resistance mutations. Of the 10 resistance-associated positions previously described in NCBI AMR and CARD databases (Table 2), four were present in all strains, including the reference strain used for alignment. This suggests that these variants represent fixed or highly conserved positions rather than markers of acquired resistance. Across the data set, a total of 104 additional mutations were detected, ranging from one to eight mutations per genome, with 77.8% (81 out of 104) present in fewer than 1% of genomes. Additionally, since *C. trachomatis* carries two copies of the 23S rRNA gene, we examined whether any mutations were heterozygous. Only 11 of the 89 complete genome assemblies exhibited heterozygous mutations, all of which were rare (present in less than 1% of genomes). To better understand lineage-specific patterns, we therefore visualized the 15 most frequent mutations. The 15 most prevalent mutations were non-homoplasious and largely lineage-specific, with genomes from the NP-UA lineage displaying the most rare mutations. However, the observed frequencies of mutations were influenced by the use of the NP-UA type strain (NC_000117.1, D/UW-3/Cx) as the reference for mutation calling, meaning that all differences were computed relative to this sequence.

**TABLE 2:** Number of genomes derived from each data source

Source	Number of genomes
NCBI Assembly	87
AllTheBacteria	1,083
Reference genomes^*a*^	20
NCBI SRA	159
Total	1,349

^*a*^ Reference genomes from a published study (29); 271 genomes from this study are also in the AllTheBacteria database.

Collectively, these observations indicate that, similar to *rpl*V, variation in *rpl*D and 23S rRNA is largely shaped by lineage and genomic background, and there is no strong evidence for recent selection for azithromycin resistance.

### *rpl*V, *rpl*D, and 23S rRNA are not in genomic recombination hotspots

**Fig 4:**
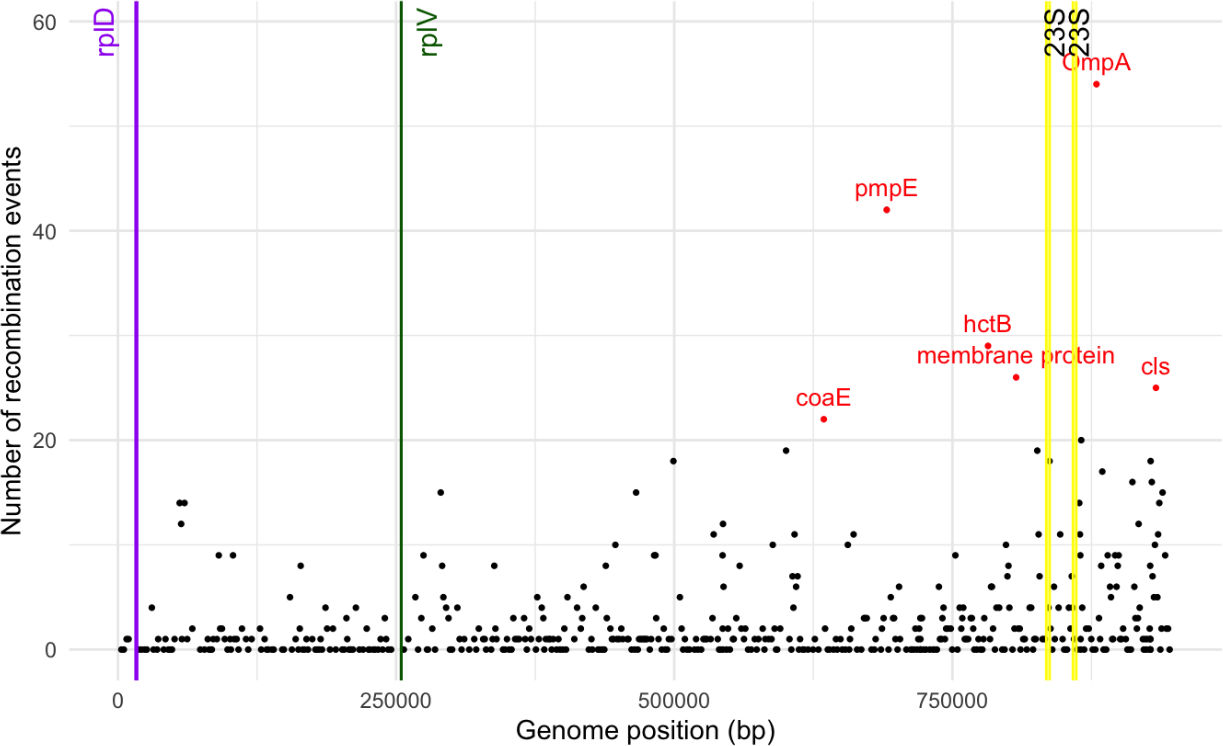
Recombination hotspots across *C. trachomatis* genome. Frequency of recombination events mapped to the *C. trachomatis* reference strain D/UW-3/Cx (NCBI Accession: NC_000117.1). The vertical lines indicate the genomic positions of the *rpl*D (purple), *rpl*V (green), and two copies of the 23S rRNA (yellow) genes. Regions with more than 20 recombination events are shown as red dots, representing recombination hotspots across the *C. trachomatis* genome.

To investigate whether resistance-associated mutations in *rpl*V, *rpl*D, or 23S rRNA arose via intra-specific recombination, we compared gene-specific phylogenies with the core-genome phylogeny for a random subset of genomes. Across all comparisons, *rpl*V vs core tree, *rpl*D vs core tree, and 23S rRNA vs core tree (Supplementary Data), we observed strong congruence, with no evidence of anomalous branching patterns indicative of horizontal transfer or recombination. The observation was further supported by genome-wide recombination analyses. None of the sequences in these ribosomal genes were located within identified recombination hotspots (Fig. 4). In contrast, recombination events were predominantly observed in expected loci, such as genes encoding membrane proteins, with *ompA* exhibiting the highest density of recombination sites.

## DISCUSSION

Many methods of inference of resistance from genome sequences require catalogs of mutations linked to phenotypes (47). Here, using 1,354 available *C. trachomatis* genomes, we investigated the phylogenetic patterns of previously characterized mutations that were purported to be linked to azithromycin resistance but varied in the degree of susceptibility or resistance based on MICs. The increasing availability of large public genomic data sets now provides an opportunity to systematically explore resistance-associated mutations across diverse populations with more comprehensive data sets. This large-scale genomic approach provides a framework for better understanding resistance dynamics in *C. trachomatis* and lays the foundation for improved surveillance and clinical management strategies.

Our study provides evidence that putative azithromycin resistance alleles were already present in *C. trachomatis* lineages before the widespread clinical use of azithromycin that occurred after 1991. The high lineage specificity of these alleles suggests that their persistence more likely reflects neutral evolution rather than resistance acquired after azithromycin use. These findings align with a growing body of work demonstrating that the genetic foundations of antimicrobial resistance often predate human antibiotic use (48), with ancient selective forces shaping the genomic background upon which modern resistance emerges (49). However, the mutations may not be directly linked to elevated resistance to azithromycin. Possibly, the putative resistance phenotype linked to *rpl*V found by other groups (19, 21) is linked to additional factors such as epistatic mutations (50). Nonetheless, these findings underscore the importance of considering historical and lineage-specific variation when interpreting the possible emergence of antibiotic resistance in *C. trachomatis*.

Pinpointing azithromycin resistance in *C. trachomatis* is complicated by several factors. Although treatment failures with azithromycin have been repeatedly reported, these events are multifactorial in nature. Reinfection from an untreated partner, inadequate drug treatment, or noncompliance with medication are common confounders that can mimic resistance-associated persistence (13, 51). Moreover, while genotypic evidence of resistance alleles provides valuable insights, it cannot be equated directly to a resistance phenotype. Several older studies highlighted this disconnect, where treatment failures were observed without clear links to resistance-associated mutations, underscoring the complexity of resistance as a trait (52). Such observations reinforce the idea that resistance in *C. trachomatis* is a multifactorial trait, with potential contributions from epistatic mutations, epigenetic regulation, host-pathogen interactions, or other yet-uncharacterized mechanisms.

Our analysis also underscores the limitations of transferring resistance markers across species. Variants annotated in public resistance databases, often curated based on other pathogens, do not consistently predict resistance in *C. trachomatis*. For example, macrolide resistance mutations in 23S rRNA (e.g., A2059G, A2074G, C2611T), which are well established in *Neisseria gonorrhoeae*, *Campylobacter coli*, and *Escherichia coli*, do not show consistent associations with resistance in *C. trachomatis*. In fact, we found these mutations in nearly all genomes in our data set, indicating that they likely reflect natural variation rather than resistance. This lack of correlation highlights the non-transferability of resistance markers between species. Instead, 23S variation in *C. trachomatis* appears to track more closely with phylogenetic background than with resistance, underscoring the need for organism-specific surveillance.

A major challenge in advancing *C. trachomatis* antimicrobial resistance research lies in the lack of phenotypic confirmation. Due to its obligate intracellular replication, traditional culture-based MIC testing is technically challenging, labor-intensive, and rarely performed in routine laboratories (53). This reliance on limited phenotypic data has constrained our ability to validate resistance mutations at scale. Nonetheless, in-depth genomic analyses, such as ours, are crucial for prioritizing candidate alleles and selecting samples for functional studies. By refining the pool of targets, these studies provide a framework to design phenotypic assays more efficiently, ultimately bridging the gap between genotypic surveillance and clinical relevance.

Taken together, our findings reinforce several key messages. First, resistance-associated alleles in *C. trachomatis* are shaped by lineage-specific evolution and likely predate clinical use of azithromycin. Second, mutations annotated as resistance markers in public databases derived from other pathogens cannot be assumed to apply to *C. trachomatis*. Third, while large-scale genomic surveys provide valuable insights into the evolutionary context of putative resistance mutations, they cannot on their own establish clinical relevance. Functional validation through phenotypic MIC testing and experimental work remains essential. Moving forward, integrating genomic surveillance with phenotypic evidence will be key to resolving the complex relationship between genetic variation and antimicrobial resistance in *C. trachomatis*, ensuring that resistance monitoring and treatment strategies are both accurate and tailored to this pathogen’s unique biology.

## SUPPLEMENTAL MATERIAL

Supplemental data aac.01708-25-s0001.docx Comparisons of core-phylogenetic tree with gene trees (RplV, RplD, and 23S rRNA); Comparisons of different BEAST models.

Supplemental tables aac.01708-25-s0002.xlsx Tables S1 to S4.

ASM does not own the copyrights to Supplemental Material that may be linked to, or accessed through, an article. The authors have granted ASM a non-exclusive, world-wide license to publish the Supplemental Material files. Please contact the corresponding author directly for reuse.
