# Supplementary material for: Putative azithromycin resistance mutations in Chlamydia trachomatis are globally distributed but arose before azithromycin was discovered
Source: Antimicrob Agents Chemother. 2026 Jan 26;70(3):e01708-25. doi: 10.1128/aac.01708-25 (PMC12959087; doi:10.1128/aac.01708-25)

Supplementary Data

## Gene-tree v/s core-tree comparisons

1. Core-tree v/s rplV tree : Randomly sub-sampled to include only 100 genomes. Comparison of the subset between core-genes tree and the single gene rplV tree.


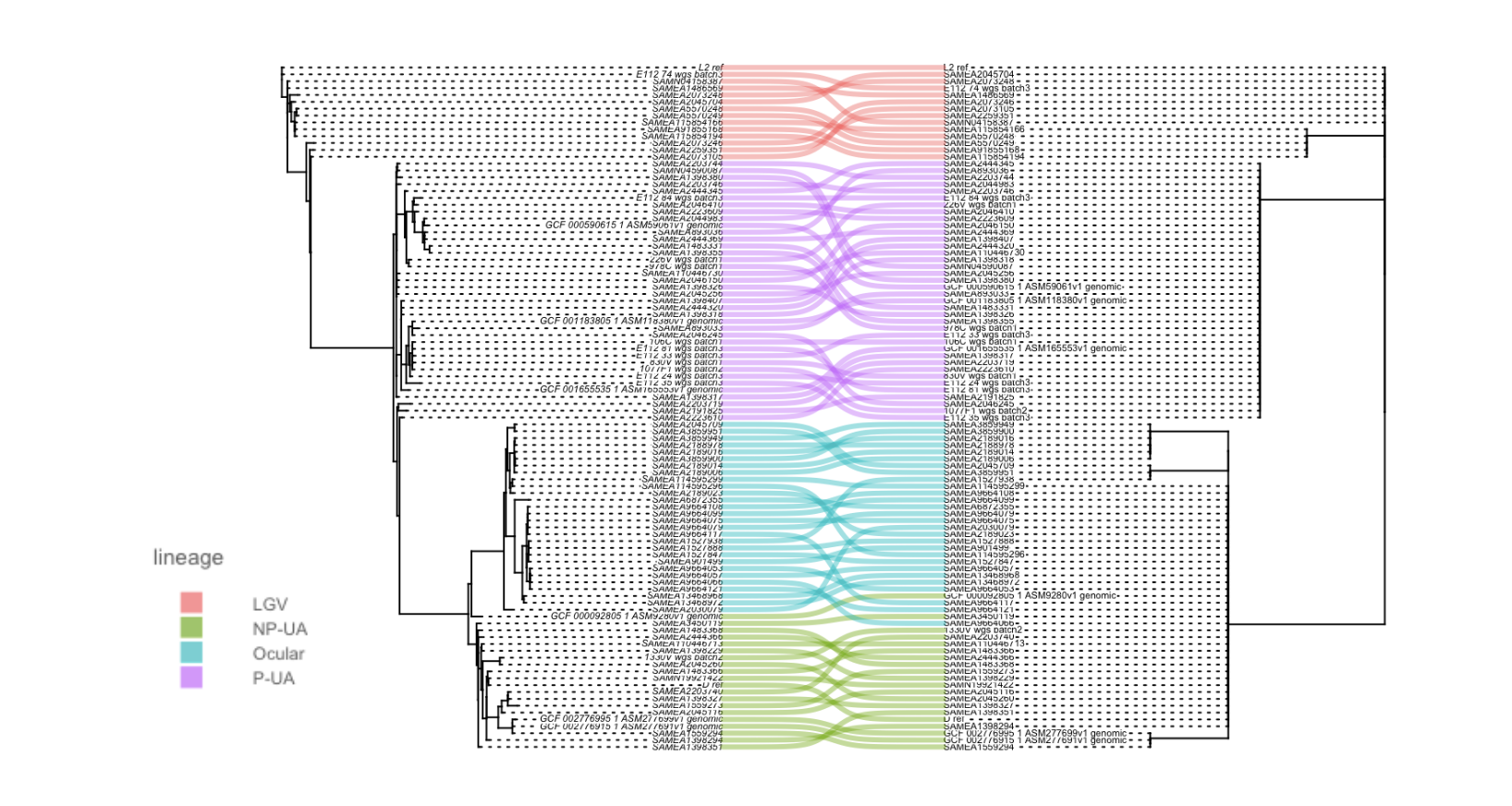


1. Core-tree v/s 23S tree:


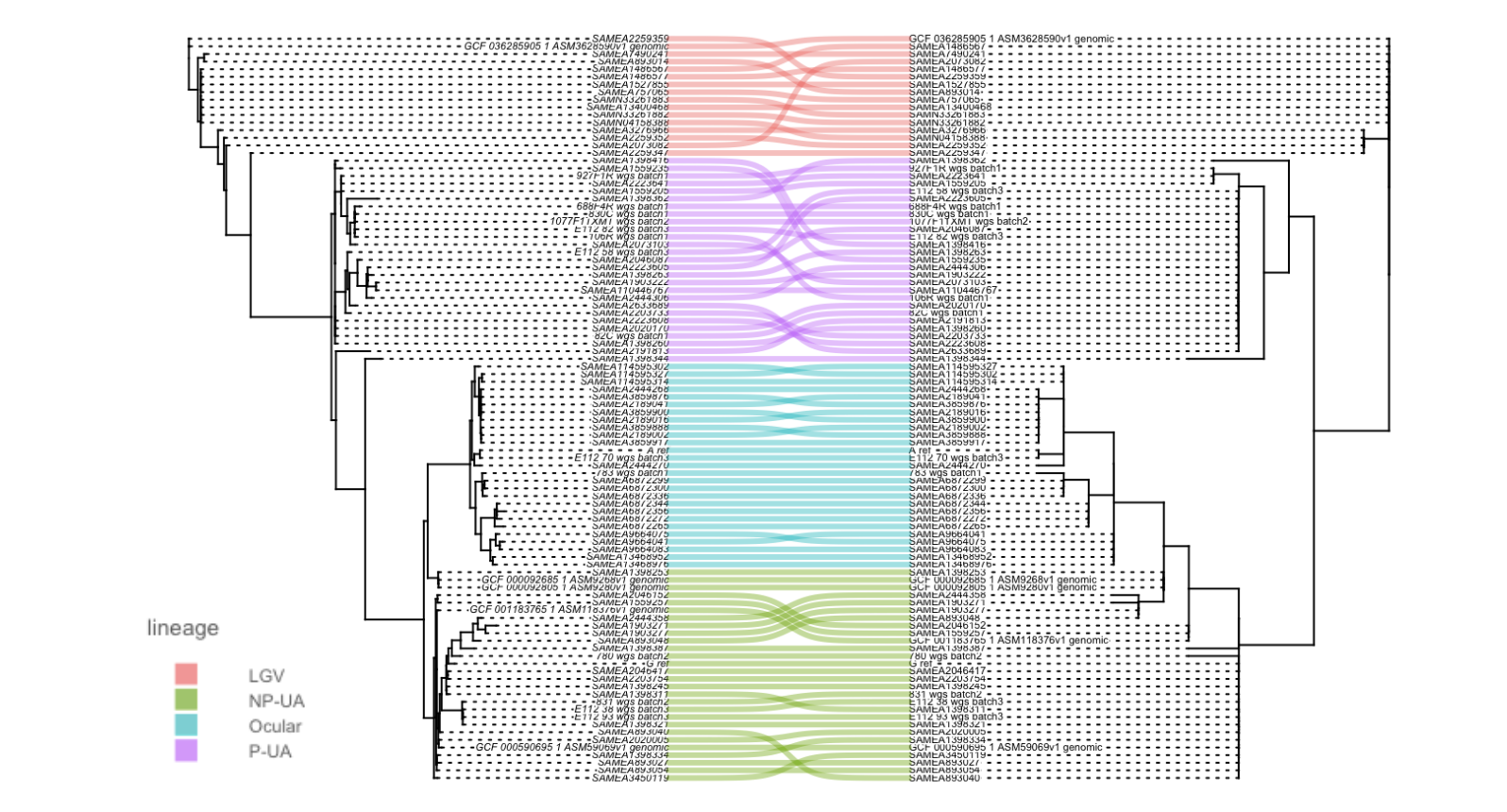


1. Core-tree v/s rplD tree:


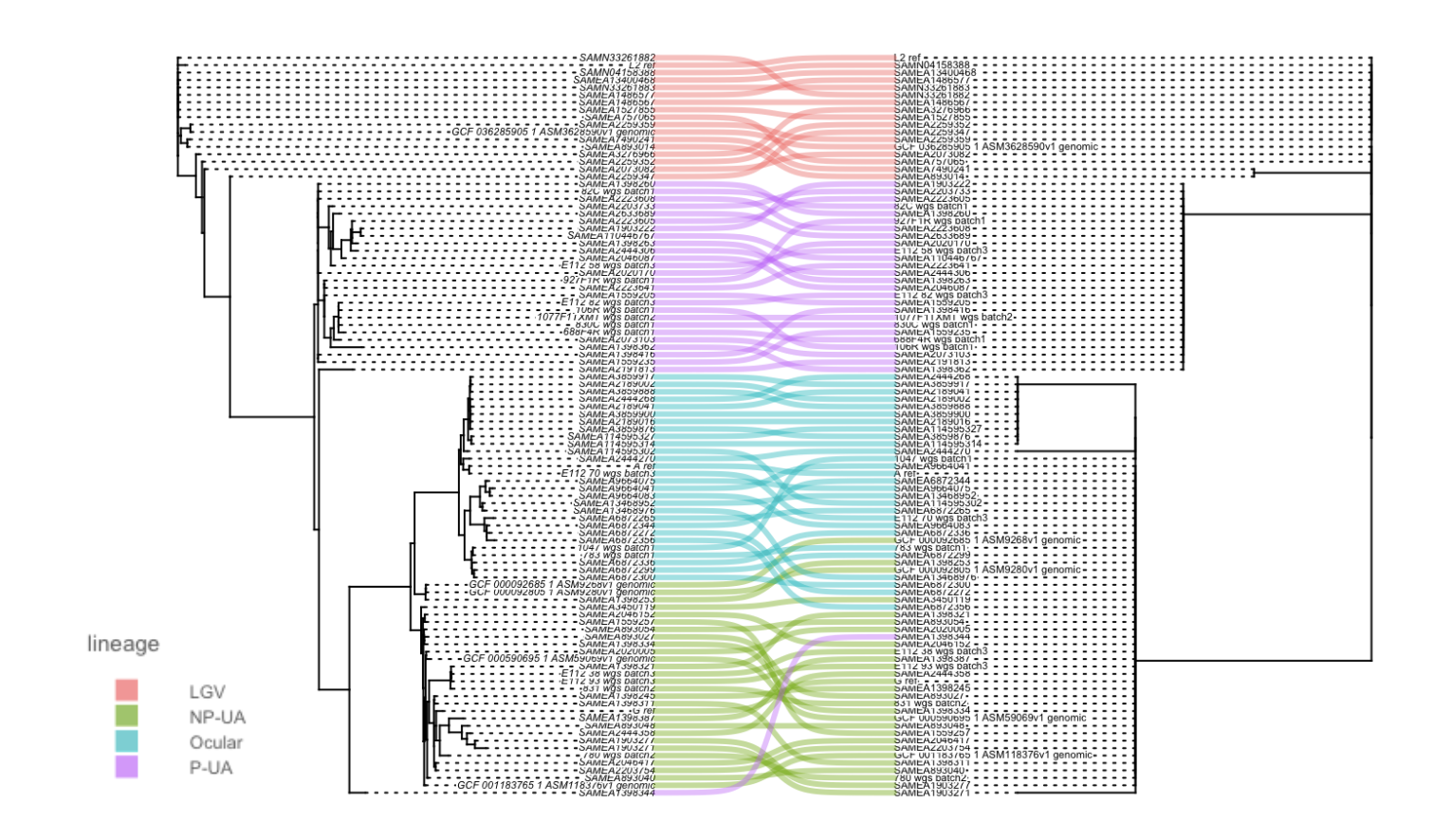


## Time-scale phylogenetic analysis : Comparison of different BEAST models

The initial dated-tree is created first using iqtree using the following command

''iqtree -s dated_alignment.fasta --date meta.tsv''

Then the resulting treefile is used as input for BEAST loosely based on the instructions in this tutorial:using the instructions form this tutorial - file:///Users/parulsharma/Downloads/DivergenceDatingTutorialv2.2.0.pdf

Different models were tried with the following parameters

1. Clock model: Strict molecular clock; Population model: Bayesian Skyline; Substitution model: GTR + Γ (4 categories)


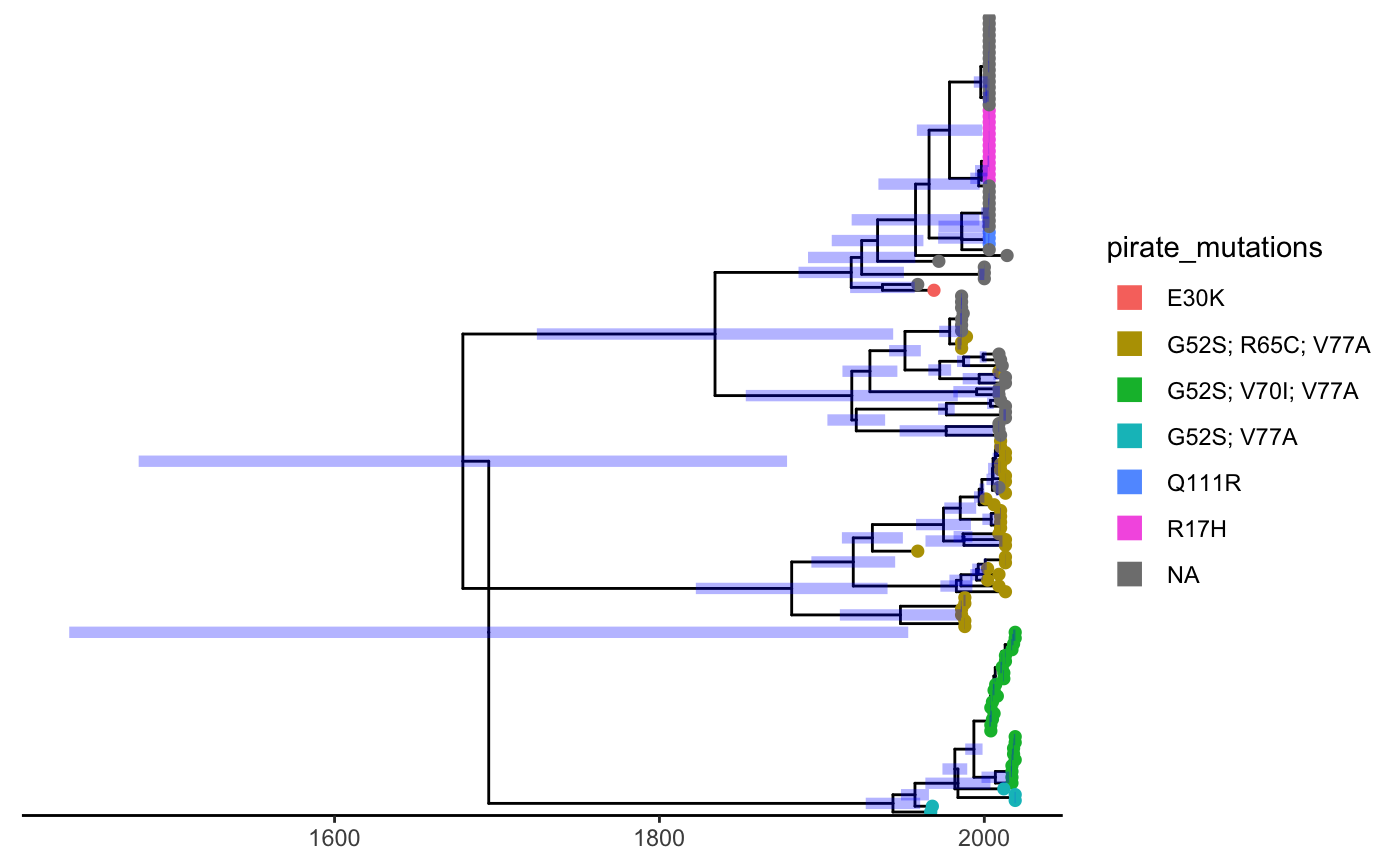


1. Clock model: Strict molecular clock; Population model: Bayesian Skyline; Substitution model: HKY + Γ (4 categories)

*HKY substitution model results in branch lengths relative to the MRCA. Hence the x-axis is not in years.


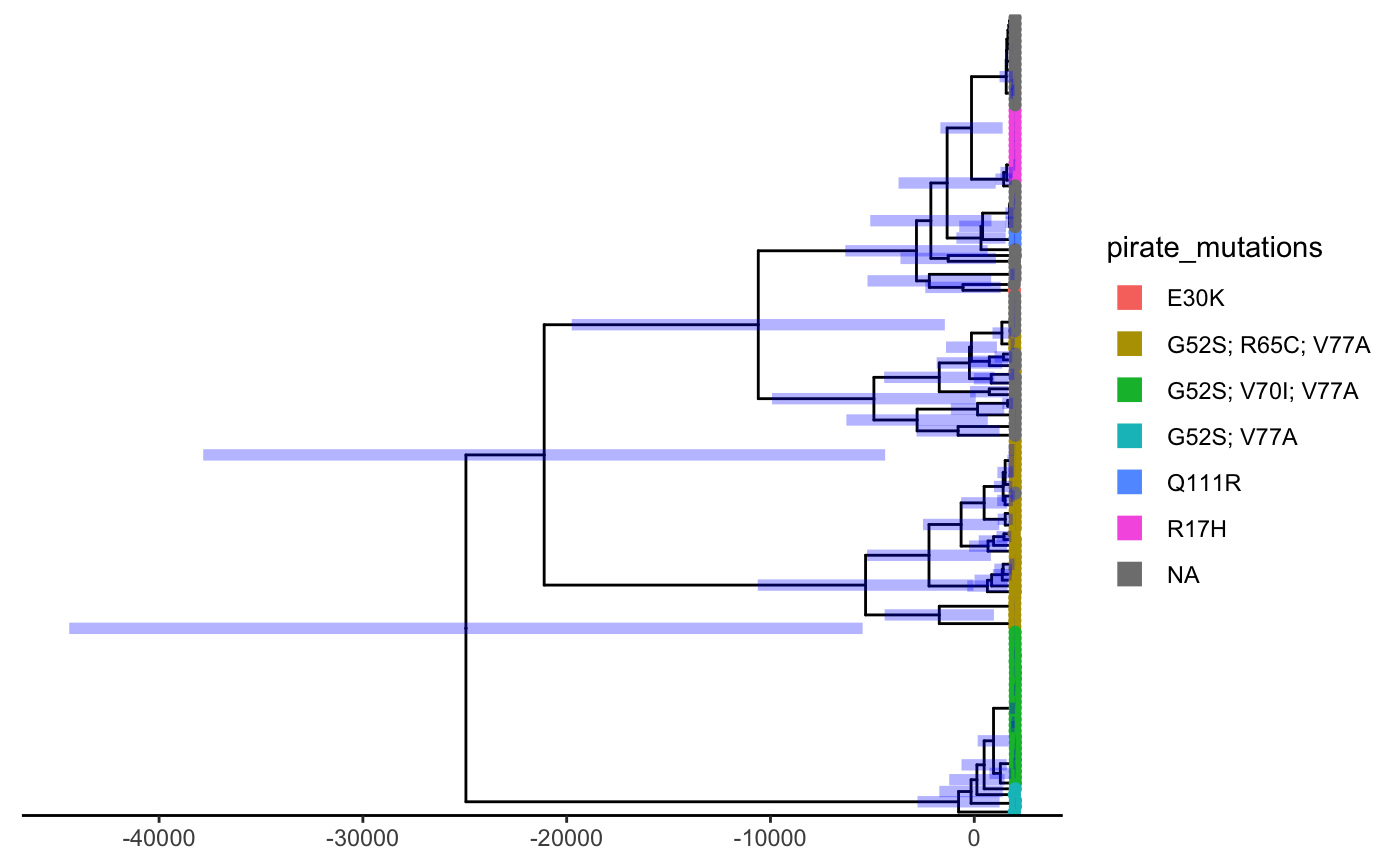


1. Clock model: Strict molecular clock; Population model: Exponential growth; Substitution model: GTR + Γ (4 categories)


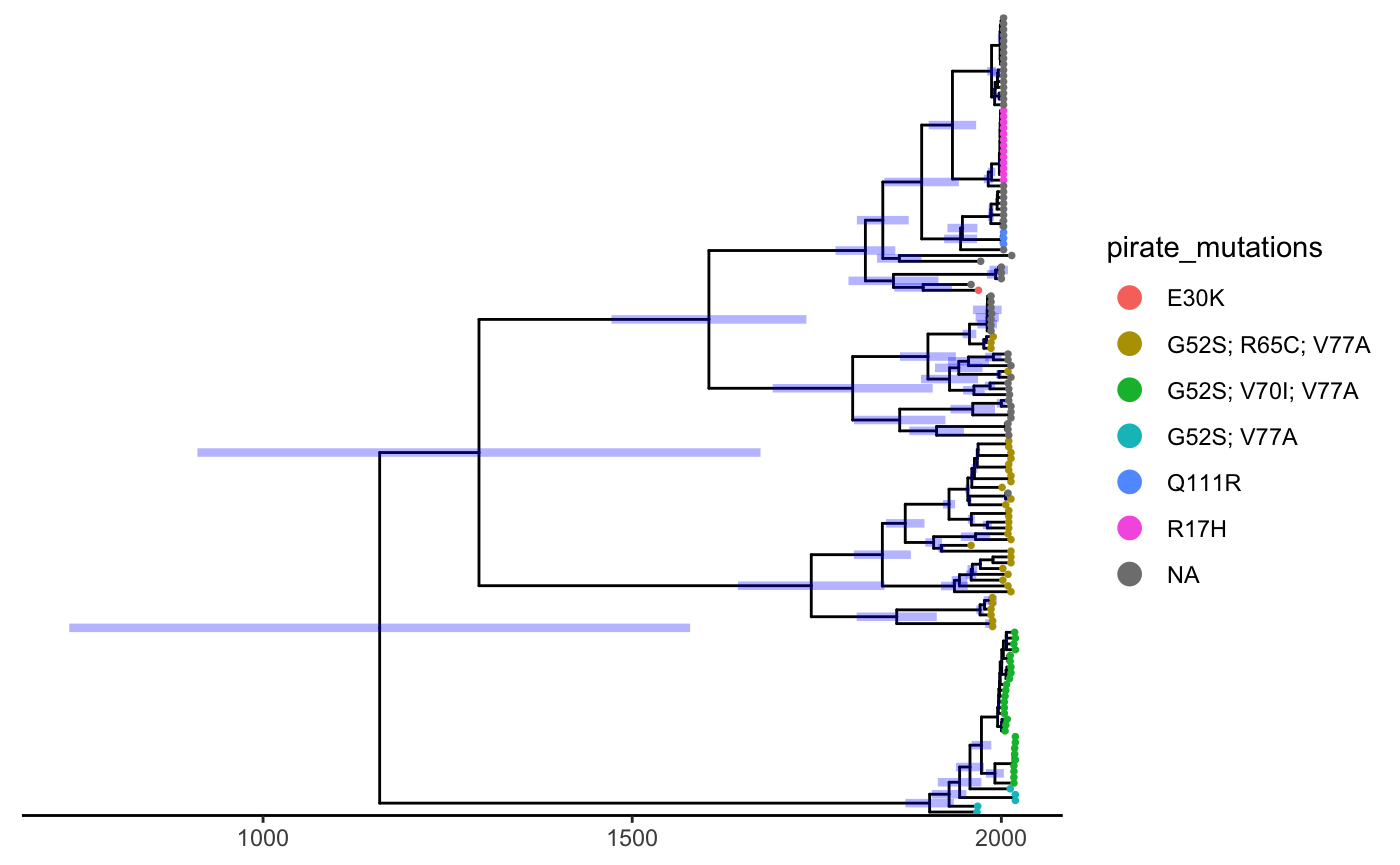


1. Clock model: Strict molecular clock; Population model: Constant size; Substitution model: GTR + Γ (4 categories)


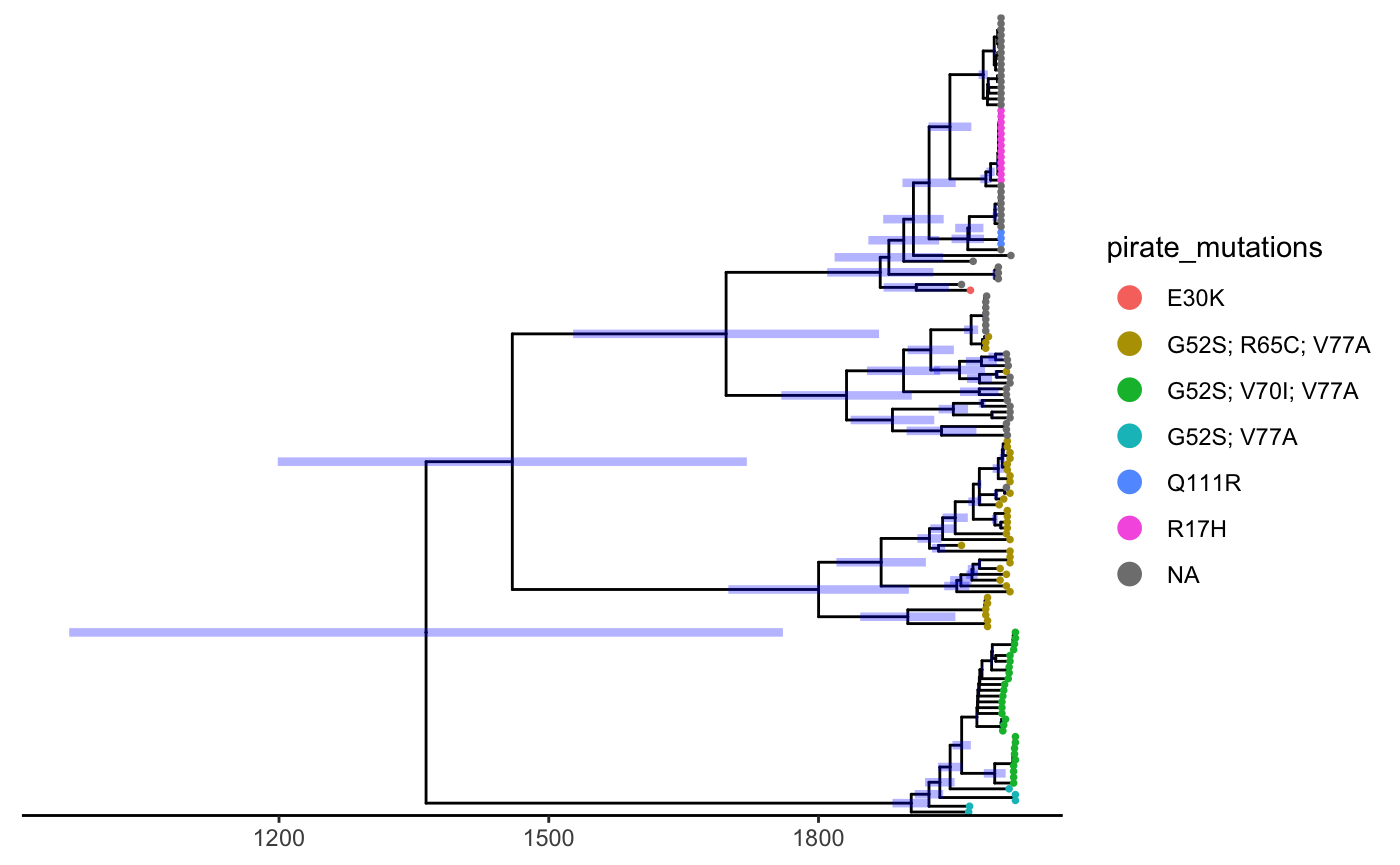


1. Clock model: Strict molecular clock; Population model: Extended Bayesian Skyline; Substitution model: GTR + Γ (4 categories+ invariant sites)


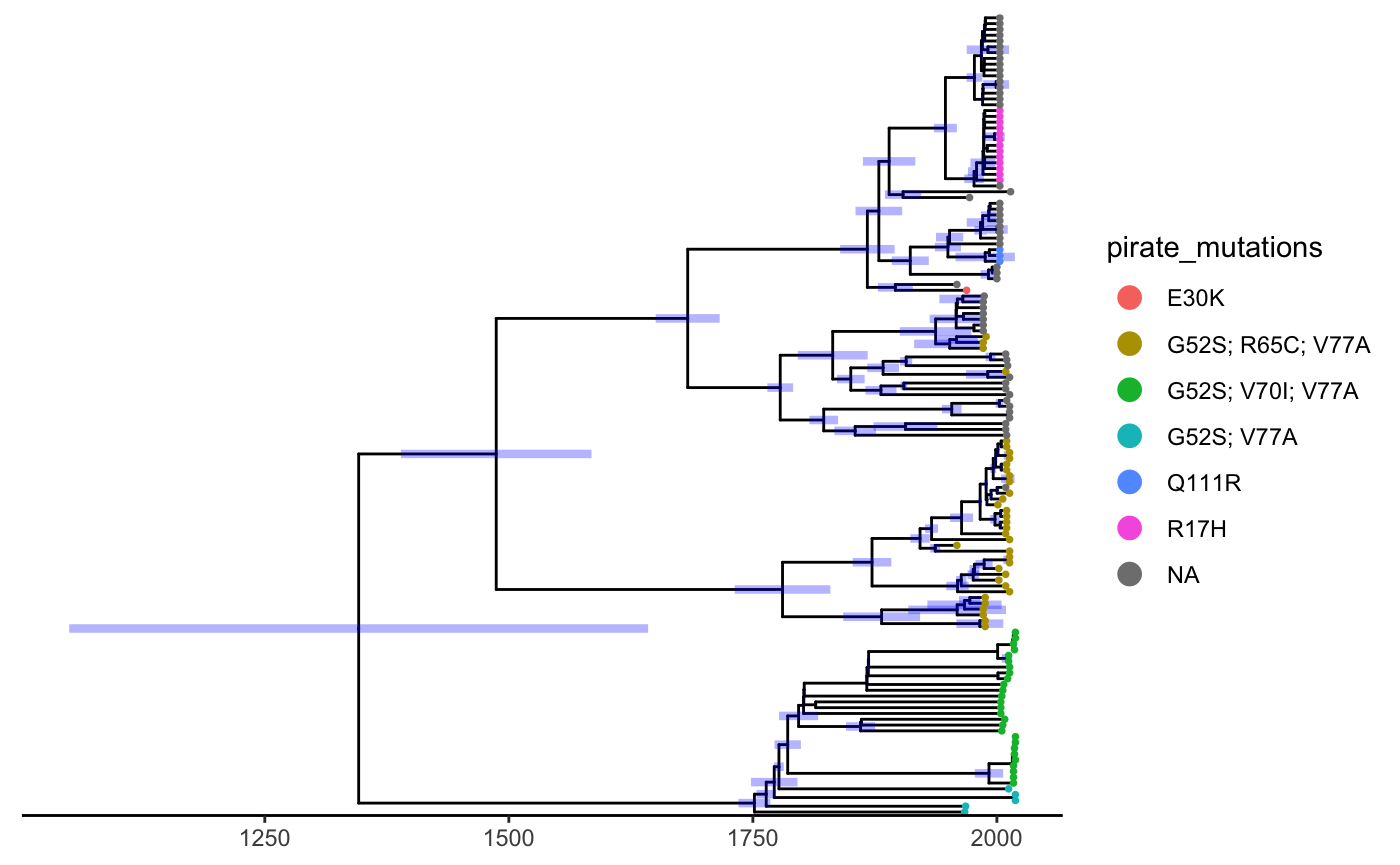

Supplement: Supplemental data — Comparisons of core-phylogenetic tree with gene trees (RplV, RplD, and 23S rRNA); Comparisons of different BEAST models. [file aac.01708-25-s0001.docx]
